# Piperonylic Acid Promotes Hair Growth by Activation of EGFR and Wnt/β-Catenin Pathway

**DOI:** 10.3390/ijms251910774

**Published:** 2024-10-07

**Authors:** Seung Hyun Han, Kyung Won Jo, Younghyun Kim, Kyong-Tai Kim

**Affiliations:** 1 Hesed Bio Corporation, Pohang 37673, Republic of Korea; jkw@hesedbio.com (K.W.J.); ted@hesedbio.com (Y.K.); 2 Generative Genomics Research Center, Global Green Research & Development Center, Handong Global University, Pohang 37554, Republic of Korea

**Keywords:** hair growth, piperonylic acid, Wnt/β-catenin, EGFR

## Abstract

Dermal papilla cells (DPCs) are located at the bottom of the hair follicle and play a critical role in hair growth, shape, and cycle. Epidermal growth factor receptor (EGFR) and Wnt/β-catenin signaling pathways are essential in promoting keratinocyte activation as well as hair follicle formation in DPCs. Piperonylic acid is a small molecule that induces EGFR activation in keratinocytes. However, the effects of piperonylic acid on DPCs in regard to the stimulation of hair growth have not been studied. In the present study, piperonylic acid was shown to activate the Wnt/β-catenin signaling pathway in addition to the EGFR signaling pathway in DPCs. Piperonylic acid suppressed DKK1 expression, which presumably promoted the accumulation of β-catenin in the nucleus. In addition, piperonylic acid promoted cyclin D upregulation and cell growth and increased the expression of alkaline phosphatase (ALP), a DPC marker. In a clinical study, the group that applied a formulation containing piperonylic acid had a significantly higher number of hairs per unit area than the placebo group. These results identify piperonylic acid as a promising new candidate for hair loss treatment.

## 1. Introduction

A dermal papilla is a small, finger-like structure located at the base of a hair follicle that plays a critical role in hair growth and maintenance. Dermal papilla cells (DPCs) produce signaling molecules that promote hair growth and increase blood flow to the hair follicle [1,2,3]. If DPCs are damaged or their functions are impaired, hair loss can occur. DPCs are mesenchyme-derived cells in the hair follicle that directly interact with epithelial cells to regulate hair growth and degeneration. Particularly, the interaction between DPCs and epithelial cells is essential for determining the hair cycle. Anagen is the most active phase of hair growth, during which DPCs send concentrated signals to the keratinocytes to generate the hair shaft [1,4]. Catagen starts after the end of anagen, and the hair growth stops and the hair follicle shrinks. The connection between DPCs and keratinocytes is severed during this stage, which typically lasts for approximately 2 to 3 weeks. Telogen is the resting phase of the hair growth cycle, and the hair is no longer attached to the DPC. The hair follicle remains in the telogen phase for approximately 2 to 3 months and then restarts the cycle by entering a new anagen phase [5,6].

Wnt/β-catenin is an essential signaling pathway in hair growth, promoting the proliferation and migration of hair follicle stem cells, hair matrix cells, and DPCs [7,8,9]. Activation of the Wnt/β-catenin pathway promotes the transition from telogen to anagen and extends the duration of the anagen phase [10,11,12]. The Wnt/β-catenin pathway is regulated by a complex network of signaling proteins and other factors. FZD and LRP5/6, which are located in the plasma membrane, play a role in receiving external signals [13,14]. In the cytoplasm, proteins such as APC, AXIN, casein kinase 1 (CK1), and GSK3β form a destruction complex that regulates the degradation of β-catenin [15,16]. In the nucleus, β-catenin binds to TCF/LEF to regulate the transcriptional activity of genes involved in cell proliferation [17,18]. Thus, activation of the Wnt/β-catenin signaling pathway has attracted the attention of many researchers and pharmaceutical companies as a therapeutic strategy for alopecia [12,19,20]. Similarly, an antagonist of a Wnt inhibitor could be an attractive hair loss treatment strategy [21]. The protein DKK1 is a negative regulator of the Wnt/β-catenin signaling pathway and reported to inhibit hair growth [22,23,24]. Genetic inhibition of DKK1 expression, which is reported to be helpful for hair growth, was investigated in previous reports [25,26]. However, a drug that can safely and effectively activate the Wnt/β-catenin signaling pathway has not been developed.

The importance of the epidermal growth factor receptor (EGFR) signaling pathway in DPCs has been well studied. The EGFR signaling pathway plays a critical role in cell growth, proliferation, and differentiation by activating ERK and AKT [27]. EGF promotes the proliferation of DPCs from mink by increasing the expression of notch genes [28]. Epiregulin, a member of the EGF family, activates DPCs by stimulating ErbB4 [29]. In addition, the activation of ERK or AKT has been verified to be directly involved in promoting the activity and proliferation of DPCs [30,31,32,33,34].

Piperonylic acid is a natural compound and a component in black pepper that has anti-inflammatory and anticancer effects by inhibiting the activity of lipoxygenase and has antioxidant effects by inhibiting α-glucosidase [35,36]. We recently reported that piperonylic acid promotes the proliferation and growth of keratinocytes through the activation of EGFR [37]. In addition, piperonylic acid has been reported to accelerate wound healing and to increase the expression of genes involved in wound healing in animal models [38]. However, the effects of piperonylic acid on DPCs and hair growth have not been studied. Through these previous studies, we hypothesized that piperonylic acid may be able to promote the proliferation and growth of DPC by activating the EGFR signaling pathway in DPC as it did in keratinocytes.

In the present study, we identified that piperonylic acid activates the Wnt/β-catenin and EGFR signaling pathways in DPCs and promotes growth and survival of DPCs. In addition, a human skin application study revealed that a formula containing piperonylic acid significantly enhanced hair growth compared with a formula without piperonylic acid. These results indicate that piperonylic acid promotes hair growth by effectively increasing the growth and activity of DPCs.

## 2. Results

### 2.1. Piperonylic Acid Activates the EGFR Signaling Pathway in DPCs

**Figure 1:**
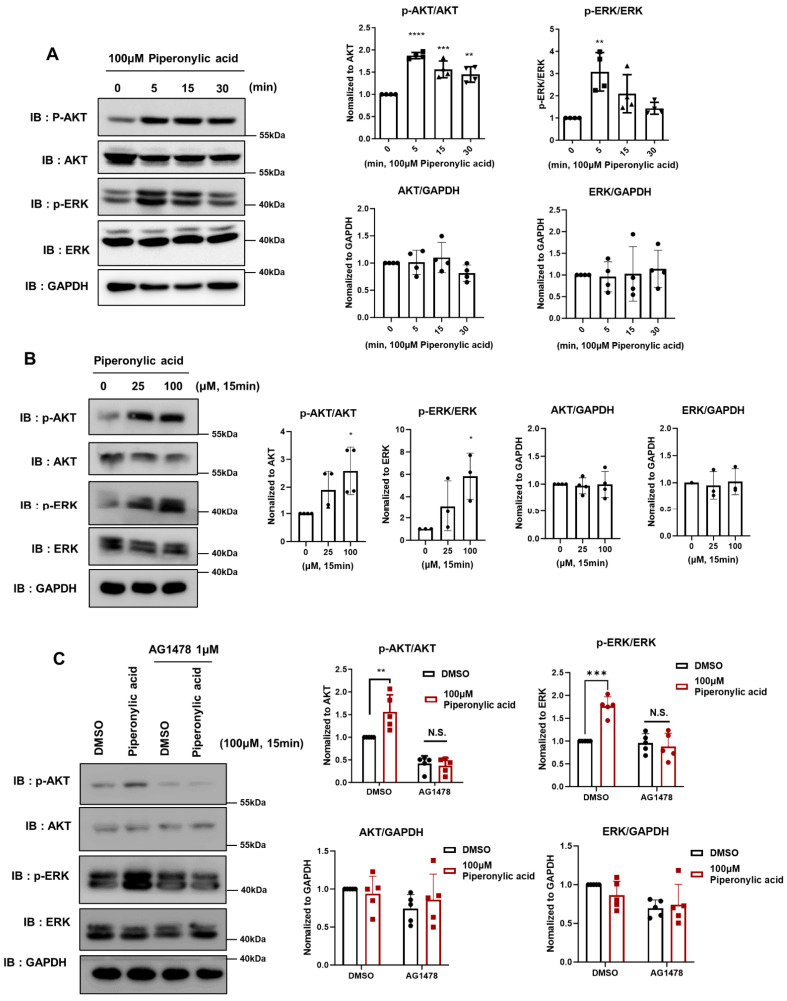
Piperonylic acid activates the EGFR signaling pathway in dermal papilla cells (DPCs). (A) Piperonylic acid increases the activity of EGFR downstream modulators in a time-dependent manner in DPCs. KNU201 was cultured in serum free media for 18 h. After incubation, the cells were treated with 100 μM piperonylic acid for 0, 5, 15, or 30 min. Cell lysates were analyzed by immunoblotting. p-AKT levels were normalized to total AKT and p-ERK levels were normalized to total ERK. Values represent mean ± standard deviation (SD) (n = 4, independent experiments). ** *p* < 0.01, *** *p* < 0.005, **** *p* < 0.001 by Tukey’s multiple comparison test. (B) Piperonylic acid increases the activity of EGFR downstream modulators in a concentration-dependent manner. KNU201 cells were cultured in serum free media for 18 h. After incubation, the cells were treated with the indicated concentration of piperonylic acid for 15 min. The cell lysates were analyzed by immunoblotting. p-AKT levels were normalized to total AKT and p-ERK levels were normalized to total ERK. Values represent mean ± standard deviation (SD) (n = 3~4, independent experiments). * *p* < 0.05 by Tukey’s multiple comparison test. (C) Piperonylic acid-induced activation of AKT was inhibited by AG1478, an EGFR inhibitor. KNU201 cells were cultured in serum free media for 8 h. After incubation, the cells were pre-treated with 1 μM AG1478 for 15 min and then treated with DMSO or 100 μM piperonylic acid for 15 min. Cell lysates were analyzed by immunoblotting. p-AKT levels were normalized to total AKT. Values represent mean ± standard deviation (SD) (n = 5, independent experiments). ** *p* < 0.01, *** *p* < 0.005 by Tukey’s multiple comparison test.

Piperonylic acid was reported to activate the EGFR signaling pathway in the HACAT keratinocyte cell line; however, its role in DPCs remains to be determined [37]. Therefore, we first tested whether piperonylic acid can regulate the EGFR signaling pathway in KNU201, which is an immortalized DPC cell line. We examined whether it activates AKT and ERK as downstream targets of EGFR by measuring the increase in the phosphorylation level of AKT and ERK. Piperonylic acid treatment significantly activated AKT and ERK in a time- and concentration-dependent manner in KNU201 cells. The expression level of AKT and ERK was not affected by piperonylic acid (Figure 1A,B). Selective EGFR blockade by AG1478, an EGFR inhibitor, abrogated piperonylic acid-mediated AKT and ERK phosphorylation, suggesting that piperonylic acid activates AKT and ERK through an EGFR-dependent pathway (Figure 1C). Also, piperonylic acid induced the activation of both AKT and ERK signaling pathways in human primary DPCs (pDPCs; Figure S1A). In summary, piperonylic acid activates the EGFR signaling pathway in KNU201 cells and pDPCs.

### 2.2. Piperonylic Acid Promotes the Activation of the Wnt/β-Catenin Signaling Pathway and Decreases DKK1

**Figure 2:**
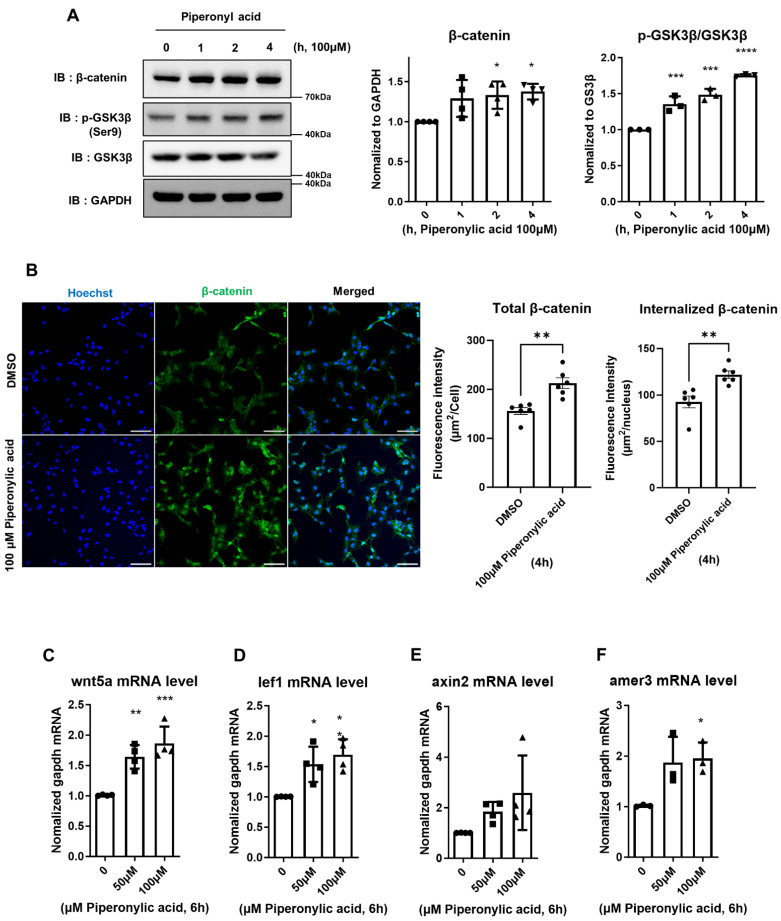

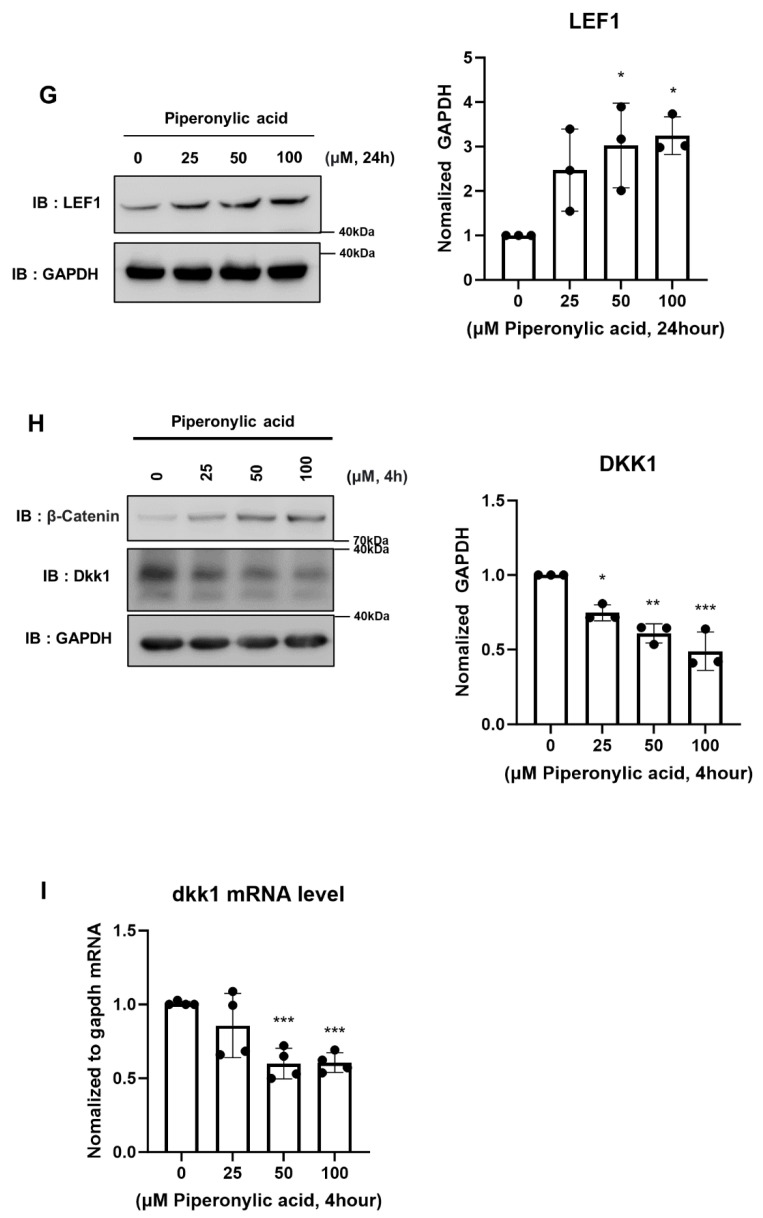
Piperonylic acid down-regulates DKK1 and activates the Wnt/β-catenin signaling pathway. (**A**) Piperonylic acid activates the Wnt/β-catenin signaling pathway in DPCs. KNU201 cells were treated with 100 μM piperonylic acid for the indicated times. Cell lysates were analyzed by immunoblotting. β-catenin levels were normalized to GAPDH and p-GSK3β(Ser9) levels were normalized to total GSK3β. Values represent mean ± standard deviation (SD) (n = 4, independent experiments). * *p* < 0.05, *** *p* < 0.005, **** *p* < 0.001 by Tukey’s multiple comparison test. (**B**) Piperonylic acid induces the accumulation of β-catenin in the nucleus of KNU201 cells. Total intensity and nuclear localization of β-catenin in response to 100μM piperonylic acid were observed by confocal microscopy. Values represent mean ± standard error of mean (SEM) (n = 6 per group, 83~149 cells per image were counted). ** *p* < 0.01 by Student’s t-test. (**C**–**F**) Piperonylic acid promotes the expression of Wnt-related genes in DPCs. KNU201 cells were treated with the indicated concentration of piperonylic acid for 6 h. mRNA levels of *WNT5A* (C), *LEF1* (D), *AXIN2* (E) and *AMER3* (F) were examined by real time PCR and normalized to *GAPDH* mRNA. Values represent mean ± standard deviation (SD) (n = 3 or 4, independent experiments) * *p* < 0.05, ** *p* < 0.01, *** *p* < 0.005. (**G**) Piperonylic acid increases the level of LEF1. KNU201 cells were treated with the indicated concentration of piperonylic acid for 24 h. Cell lysates were analyzed by immunoblotting. LEF1 levels were normalized to GAPDH. Values represent mean ± standard deviation (SD), (n = 3, independent experiments). * *p* < 0.05 by Tukey’s multiple comparison test. (**H**) Piperonylic acid decreases the level of DKK1 in DPCs. KNU201 cells were treated with the indicated concentration of piperonylic acid for 4 h. Cell lysates were analyzed by immunoblotting. DKK1 levels were normalized to GAPDH. Values represent mean ± standard deviation (SD), (n = 3, independent experiments). * *p* < 0.05, ** *p* < 0.01, *** *p* < 0.005 by Tukey’s multiple comparison test. (**I**) Piperonylic acid decreases the expression of DKK1 in DPCs. KNU201 cells were treated with the indicated concentration of piperonylic acid for 4 h. mRNA level of DKK1 was examined by real time PCR and normalized to GAPDH mRNA. Values represent mean ± standard deviation (SD) (n = 4, independent experiments) *** *p* < 0.005, by Tukey’s multiple comparison test.

Crosstalk between the EGFR and the Wnt/β-catenin signaling pathway is widely reported [39,40,41]. Therefore, we investigated whether piperonylic acid affects the Wnt/β-catenin signal along with the EGFR signaling pathway. Phosphorylation of GSK-3β leads to the accumulation of β-catenin in the nucleus and activates the Wnt/β-catenin signaling pathway. Notably, treatment with piperonylic acid caused a significant increase in both phosphorylation of GSK-3β at the serine 9 (ser9) residue and β-catenin levels in KNU201 cells (Figure 2A). Immunocytochemistry showed an increased total β-catenin level and a marked increase in its nuclear localization when KNU201 cells were treated with piperonylic acid (Figure 2B). AG1478 treatment did not modulate piperonylic acid-induced β-catenin accumulation, indicating that piperonylic acid acts independently of the EGFR pathway (Figure S2A). Consistent with the trend of Wnt/β-catenin signaling pathway activation, piperonylic acid upregulated the mRNA levels of Wnt-associated genes in KNU201 cells, including *WNT5A*, *LEF1*, and *AMER3* [42,43,44]. *AXIN2* also showed an increasing trend upon piperonylic acid treatment, but it was not statistically significant (Figure 2C–F). A concentration-dependent increase was observed in the LEF1 protein level, a well-known Wnt target (Figure 2G). In addition to KNU201, treatment with piperonylic acid induced an increase in p-GSK3β and β-catenin levels as well as Wnt target genes in pDPCs derived from humans (Figure S2B–E).

Because a piperonylic acid-induced alteration was observed in p-GSK3β and β-catenin, we hypothesized that piperonylic acid may have a modulatory role upstream of the Wnt/β-catenin signaling cascade. Among the regulators of Wnt/β-catenin signaling, we focused on DKK1, which binds to LRP5/6 and strongly inhibits the activity of the Wnt/β-catenin pathway. DKK1 is also induced by the androgen receptor (AR) signaling pathway and has negative effects on hair growth [24]. We tested whether piperonylic acid regulates the DKK1 level. Western blot and qPCR results showed that piperonylic acid treatment significantly inhibited DKK1 expression at 4 h of treatment in a concentration-dependent manner (Figure 2H,I). Furthermore, DKK1 expression continued to be inhibited by piperonylic acid after 24 h of treatment (Figure S2F). Thus, we tested whether piperonylic acid is associated with androgen signaling. Although piperonylic acid induced downregulation of DKK1, changes were not observed in the mRNA expression of AR or IL-6, which are also target genes of AR in DPCs. This result suggests that DKK1 modulation by piperonylic acid is independent of the AR signaling pathway [45] (Figure S2G,H). In summary, piperonylic acid effectively reduces DKK1, a negative regulator of Wnt, and activates the Wnt/β-catenin signaling pathway in DPCs.

### 2.3. Piperonylic Acid Promotes the Induction of Alkaline Phosphatase (ALP)

**Figure 3:**
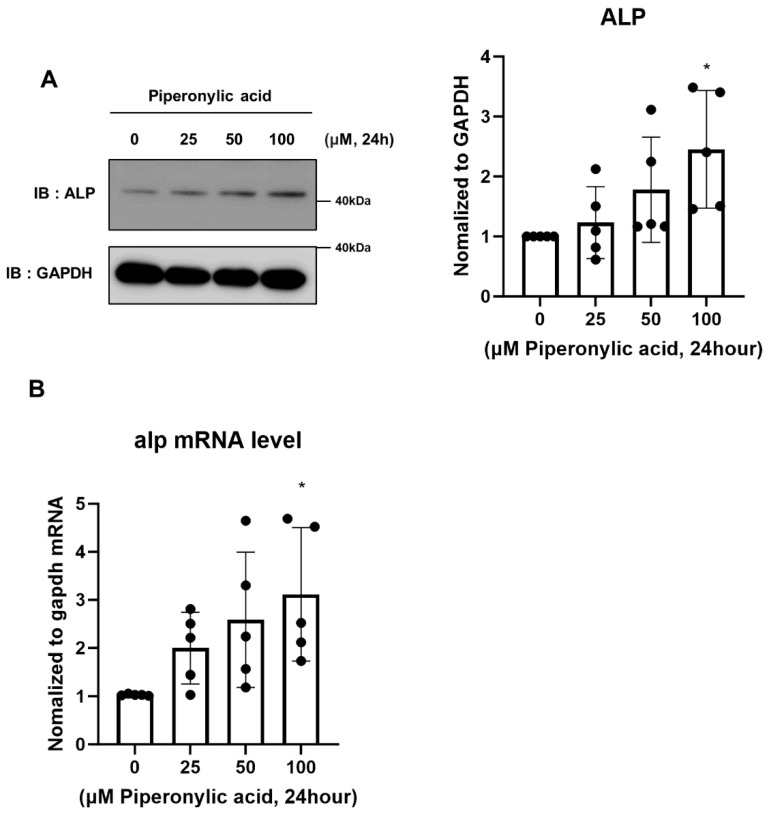
Piperonylic acid increases the level of ALP. (A) Piperonylic acid induces expression of ALP in DPCs. KNU201 cells were treated with the indicated concentrations of piperonylic acid for 24 h. Cell lysates were analyzed by immunoblotting. ALP levels were normalized to GAPDH. Values represent mean ± standard deviation (SD) (n = 5, independent experiments). * *p* < 0.05 by Tukey’s multiple comparison test. (**B**) Piperonylic acid increases the expression of *alp* in DPCs. KNU201 cells were treated with the indicated concentrations of piperonylic acid for 24 h. *ALP* mRNA was examined by real time PCR and normalized to *GAPDH* mRNA. Values represent mean ± standard deviation (SD) (n = 5, independent experiments) * *p* < 0.05, by Tukey’s multiple comparison test.

Alkaline phosphatase (ALP) is a highly activated enzyme in the dermal papilla during early anagen. ALP plays a positive role in the capacity of the DPCs and the inductivity of the hair follicle [2,46,47]. Therefore, ALP is used as a biological marker to measure the activity of DPCs in various studies [17,44]. Based on the finding that piperonylic acid increases signaling pathways associated with DPC activity, we investigated whether piperonylic acid affects ALP expression. Notably, piperonylic acid treatment increased ALP mRNA and protein levels in KNU201 cells in a concentration-dependent manner (Figure 3A,B). These results suggest that piperonylic acid is a positive regulator that promotes DPC inductivity.

### 2.4. Piperonylic Acid Promotes Induction of Cell Growth-Related Factors and DPC Growth

**Figure 4:**
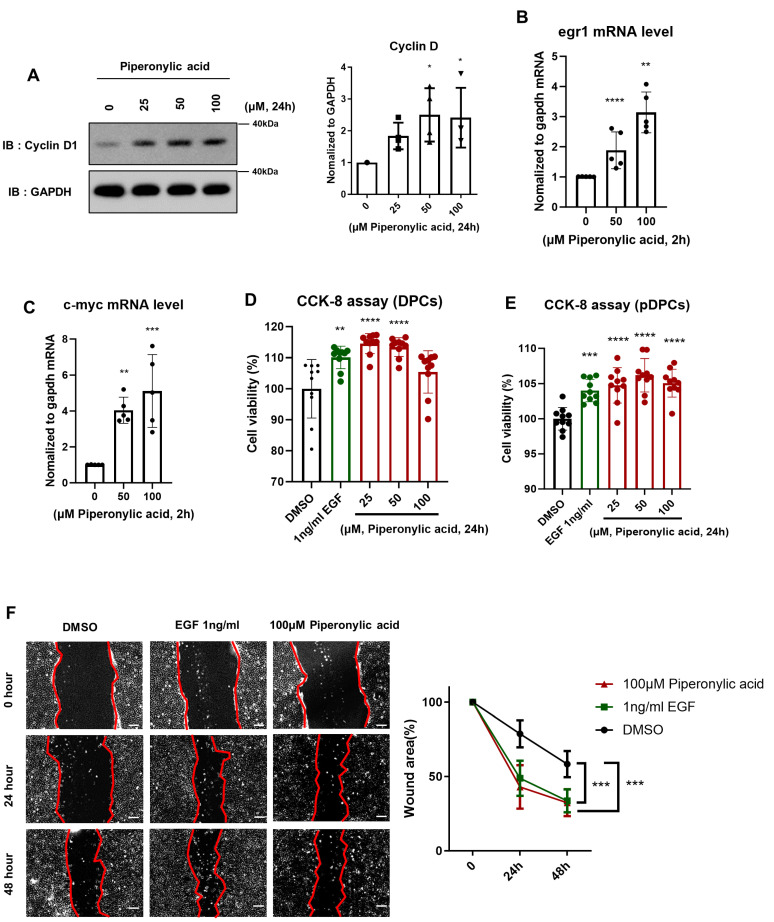
Piperonylic acid promotes the induction of cell proliferative factors and DPCs growth. (A) Piperonylic acid increases the level of cyclin D. KNU201 cells were treated with the indicated concentrations of piperonylic acid for 24 h. Cell lysates were analyzed by immunoblotting. Cyclin D levels were normalized to GAPDH. Values represent mean ± standard deviation (SD), (n = 3, independent experiments). * *p* < 0.05 by Tukey’s multiple comparison test. (**B**,**C**) Piperonylic acid increases the expression of genes related to cell growth. KNU201 cells were treated with the indicated concentrations of piperonylic acid for 2 h. mRNA levels of *EGF1* (B) and *C-MYC* (C) were examined by real time PCR and normalized to *GAPDH* mRNA. Values represent mean ± standard deviation (SD) (n = 5, independent experiments) ** *p* < 0.01,*** *p* < 0.001, **** *p* < 0.0001 by Tukey’s multiple comparison test. (**D**,**E**) Piperonylic acid promotes cell growth. (**D**) KNU201 cells were treated with 1 ng/mL of EGF or the indicated concentrations of piperonylic acid for 24 h and cell viability was detected by CCK-8. (n = 10) * *p* < 0.05, **** *p* < 0.0001 by Tukey’s multiple comparison test. (**E**) pDPCs were treated with 1 ng/mL of EGF or the indicated concentrations of piperonylic acid for 24 h and cell viability was detected by CCK-8. (n = 10) *** *p* < 0.001, **** *p* < 0.0001 by Tukey’s multiple comparison test. (**F**) Piperonylic acid promotes the healing process of DPCs. KNU201 cells were treated with 1 ng/mL of EGF or 100 μM piperonylic acid. The wound areas were measured using Image J. Values represent mean ± standard deviation (SD), (n = 7) ***p < 0.001. Two-way ANOVA followed by Tukey’s multiple comparisons test.

Cyclin D, a well-established cell cycle regulator, promotes the G1/S transition in DPCs and is a downstream target of both the EGFR and Wnt/β-catenin signaling pathways [48,49]. Piperonylic acid-induced activation of the EGFR and Wnt/β-catenin signaling pathways led to an increased cyclin D1 level. Piperonylic acid treatment elicited a concentration-dependent increase in the cyclin D1 level in KNU201 cells at 24 h of treatment (Figure 4A). In addition, piperonylic acid significantly upregulated the expression of cell growth-related genes, such as *EGR1* and *C-MYC*, in KNU201 cells (Figure 4B,C). 

Cell proliferation and migration of DPC is essential for hair growth [50,51]. Therefore, we also investigated the effects of piperonylic acid on cell proliferation and migration of KNU201 cells and pDPCs. The CCK-8 assay results showed piperonylic acid to significantly promote cell growth in both KNU201 cells and pDPCs, similar to EGF (Figure 4D,E). Significant cytotoxicity was not observed up to 100 μM. The wound healing assay showed enhanced cell proliferation and migration in piperonylic acid-treated KNU201 cells (Figure 4F). In summary, piperonylic acid promotes cell growth by inducing the expression of cyclin D1 and genes involved in cell growth in DPCs.

### 2.5. Piperonylic Acid-Containing Formula Accelerates Hair Growth in a Human Clinical Study

**Figure 5:**
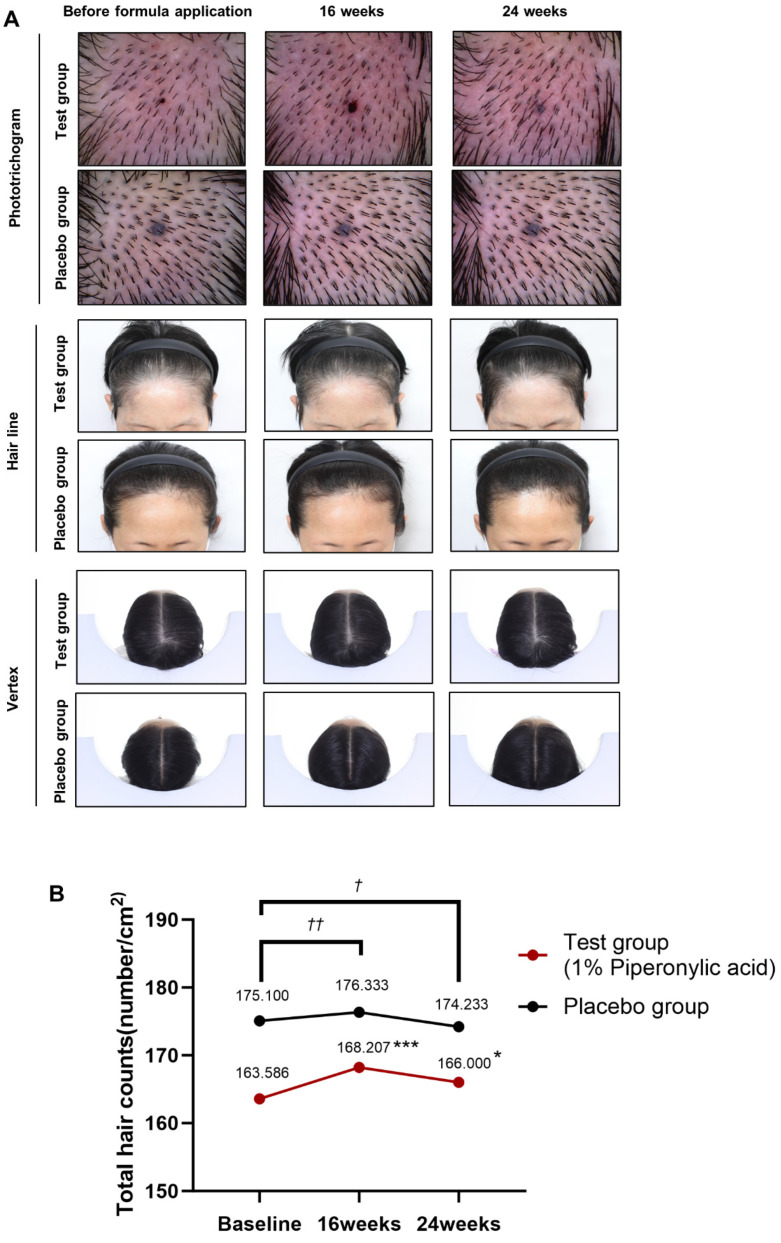
Effect of piperonylic acid in a human clinical study. (**A**) Images of the change in hair loss. Hair line, vertex and phototrichogram images at 0, 16 and 24 weeks. (**B**) Relative total hair counts. Changes in relative total hair counts at 16 and 24 weeks after formula application. Within-subject significance probability at 16 and 24 weeks compared to baseline * *p* < 0.05, *** *p* < 0.001 by repeated measures ANOVA with contrast test. The change in hair count after applying each formula for 16 weeks and 24 weeks compared to 0 week and significance probability between the test group and placebo group. ^†^ *p* < 0.05, ^††^ *p* < 0.01 by Mann–Whitney U test using delta values (Δ). Delta value (Δ) is the change in hair count between the pre-application measurement and the post-application measurement.

We aimed to investigate whether the activation and proliferation of DPCs caused by piperonylic acid are associated with hair growth in humans. Here, 59 males and females with moderate androgenic alopecia were recruited and randomly assigned to either a piperonylic acid-containing formula group (test group, n = 29) or a non-piperonylic acid-containing formula group (placebo group, n = 30; Figure 5A). A phototrichogram was used to measure the total number of hairs in the balding area. The results of the within-subject effect test and Friedman test for the number of hairs before and after applying each formula showed total hair count measurement values in the test group to be significantly increased after formula application (*p* < 0.001). Conversely, a significant difference in total hair count was not observed in the placebo group (Table 1). A within-subject contrast test also showed significantly increased hair count measurement values in the test (16 weeks (168.207 ± 29.493) *p* < 0.001, 24 weeks (166.000 ± 28.385) *p* = 0.03) compared with the starting point of the formula application (163.586 ± 30.205); however, differences were not observed in the placebo group (before applying the formula (175.100 ± 34.288), 16 weeks (176.333 ± 35.346), 24 weeks (174.233 ± 33.685); Table 2). Examination of the individual hair count from the balding area showed a significant improvement in the test group, with an average increase of 3.01% at 16 weeks and 1.767% at 24 weeks of formula application. Conversely, the placebo group showed minimal or negative changes, with an increase of 0.67% at 16 weeks and a decrease of −0.403% at 24 weeks of formula application (Table 3). The results of the pre-treatment homogeneity test showed the hair count measurements between groups were not significantly different (Table S1). Groupwise comparison of the hair count changes revealed significantly greater hair growth in the test group compared with the placebo group at both 16 weeks (*p* = 0.009) and 24 weeks (*p* = 0.047) of application (Table 4). In the safety assessment, subjects and dermatologists did not observe any adverse reactions of the skin throughout the clinical study. Based on the results of the clinical study, piperonylic acid helped promote hair growth in human subjects and could be a promising candidate for hair growth.

## 3. Discussion

Hair loss is not a life-threatening or physically painful condition but can have a negative psychological impact on individuals. In modern society, the increased interest in appearance has led to explosive growth of the hair care market. However, only two FDA-approved hair loss medications are currently available: finasteride and minoxidil [52]. Finasteride is a 5-alpha reductase inhibitor that effectively inhibits the production of DHT. However, it can cause sexual dysfunction in males and increase the risk of birth defects in the fetuses of pregnant females [53,54]. Minoxidil was developed as a treatment for hypertension; however, the drug is also effective against hair loss and is used as a topical medication. Minoxidil is relatively safe but not effective for severe hormonal hair loss and can cause scalp irritation and itching [55,56]. Despite ongoing efforts by numerous researchers to develop new hair loss treatments utilizing diverse strategies, additional therapeutic options have not achieved acceptable efficacy and safety levels. In this manuscript, piperonylic acid, a promising natural hair growth stimulator devoid of evident adverse effects, was presented. In vitro evaluations showed no cytotoxicity by piperonylic acid even at high concentrations (>100 μM), and a 1% piperonylic acid formulation showed excellent safety in a clinical study with 29 participants. In terms of efficacy, topical application of a piperonylic acid formulation resulted in enhanced hair growth compared with placebo in a randomized, double-blind clinical study.

The role of EGFR signaling in hair growth is controversial. In previous reports, EGF had a positive effect on hair growth by promoting the growth and migration of outer root sheath (ORS) cells in the hair follicle through the activation of Wnt/β-catenin [57]. EGF also promotes the growth of hair follicle-derived mesenchymal stem cells through ERK and AKT signaling [58]. In mink-derived DPCs, activation of EGFR increased the expression of the notch1 downstream genes Hes1 and Hes5, promoting the growth of DPCs [28]. In addition, a kinase-inactive EGFR knock-in mouse model was reported to exhibit defective hair follicle development compared with wild-type (WT) mice, indicating the need for EGFR activity for hair follicle development in postnatal mice [59]. The finding that epiregulin, a member of the EGF family, increases the activity of DPCs indicates that various EGFR stimuli may induce DPC activation by activating Erbb receptors [29]. Conversely, EGFR signaling has been suggested to inhibit hair growth. EGF promotes the transition to catagen by suppressing the expression of stathmin1 [60]. In the study by Kashiwagi et al., EGF, KGF, and TGF-α inhibited the formation of hair buds in the skin of embryonic day 13.5 mice [61]. In summary, these controversies indicate the variable effects of EGF on hair growth depending on the amount of EGF, the stage of hair growth cycle, and the cell type acted on. Therefore, EGF is a very delicate and complex regulator of hair growth and loss. However, the EGFR signaling pathway in adult animals appears to be primarily responsible for promoting hair growth [62].

Piperonylic acid significantly and rapidly enhanced the activity of ERK and AKT, the downstream targets of EGFR. Activation of ERK and AKT has been shown to increase DPC growth and activity in numerous studies. Therefore, the activation of EGFR signaling caused by piperonylic acid in DPCs could have a positive effect on hair growth. However, whether piperonylic acid can activate other Erbb family members in addition to EGFR has not been confirmed. In future studies, it is necessary to elucidate the Erbb receptors activated by piperonylic acid in DPCs. In this study, AG1478 was used to inhibit EGFR signaling. Although AG1478 is a well-known EGFR inhibitor, recently it was reported to have off-target effects.

Piperonylic acid promotes the phosphorylation of GSK3β, leading to its inactivation and significantly increased β-catenin. Piperonylic acid also increases the expression of various genes that contribute to hair growth. Because piperonylic acid was reported to activate EGFR, we investigated whether the increase in β-catenin induced by piperonylic acid is dependent on EGFR. However, piperonylic acid-induced accumulation of β-catenin was not decreased by AG1478, indicating that piperonylic acid acts independently on each signaling pathway. Notably, piperonylic acid inhibited DKK1 expression in DPCs, indicating piperonylic acid may promote Wnt/β-catenin signaling by suppressing DKK1 expression. However, the molecular mechanism by which piperonylic acid inhibits DKK1 expression is not fully understood, and further studies are needed to investigate how piperonylic acid regulates DKK1 expression.

Among the Wnt-associated genes upregulated by treatment with piperonylic acid, Wnt5a was also upregulated. Wnt5a was previously reported to be associated with non-canonical Wnt pathways. Moreover, there is a suggestion that claims Wnt5a is a suppressor of the Wnt/β-catenin signaling pathway, which leads to inhibition of hair growth [63]. However, in another study, we found that minoxidil treatment increased expression of Wnt5a along with increased expression of β-catenin [44]. Therefore, we considered this result to be interesting and further study is necessary to fully elucidate the role of Wnt5a in hair growth in the future.

Piperonylic acid is currently listed in the cosmetics raw materials dictionary and has been reviewed by the Personal Care Products Council (PCPC). In a clinical study, 59 subjects with androgenic alopecia were treated with a formula containing piperonylic acid or placebo formula applied twice a day to the balding area of their scalps. In the test group, the number of hairs increased by 3.01% at 16 weeks (from 163.586 ± 30.205 to 168.207 ± 29.493) and by 1.767% at 24 weeks (from 163.586 ± 30.205 to 166.000 ± 23.385). However, significant hair growth was not observed in the placebo group. Compared with the placebo formula, the test formula significantly enhanced the average hair count by 4.621 hairs (*p* = 0.009) at 16 weeks and 2.414 hairs (*p* = 0.047) at 24 weeks. However, the placebo group showed an average hair loss of 0.867 hairs at 24 weeks. According to these results, the formula may seem to be more effective at 16 weeks and its effect seems to weaken at 24 weeks of treatment, suggesting limited duration of the effect of piperonylic acid. However, the reason for this result cannot be fully elucidated with the current results that are available. Perhaps these results could be temporary because the hair cycle of each subject in a clinical trial is different. Since this clinical trial checked the hair growth of participants at only two time points, clinical trials that extend longer than 24 weeks may be necessary in the future to thoroughly evaluate the long-term effect of a piperonylic acid formula in hair growth of human participants. To confirm the long-term effect of the piperonylic acid formula, a clinical trial that covers about an year needs to be conducted in the future. Most subjects were satisfied with the use of the product and indicated they would purchase it again. However, because the sample size of the clinical test was small, analyses based on sex or age could not be conducted. In addition, the study was not conducted with subjects from diverse ethnicities. Therefore, future studies should be conducted with larger populations with diverse ethnicity to confirm the effectiveness of piperonylic acid across sex, ages, and racial/ethnic groups.

## 4. Materials and Methods

### 4.1. Cell Culture

The human dermal papilla cell line KNU201 was purchased from Epibiotech (Inchun, South Korea). Human primary dermal papilla cells were purchased from the Industry–University Cooperation Foundation of Kyungpook National University (Daegu, South Korea). KNU201 and pDPCs were cultured in Dulbecco’s modified Eagle’s medium (DMEM) with high glucose (Hyclone, Amersham, UK) supplemented with 10% fetal bovine serum (FBS) (Hyclone, Amersham, UK) and 1% penicillin–streptomycin (Welgene, Gyeongsan, Republic of Korea) in a humidified 5% CO_2_ incubator at 37 °C.

### 4.2. Chemicals and Antibodies

Piperonylic acid, AG1478 and Hoechst 33342 were purchased from Sigma–Aldrich (Saint Louis, MO, USA). Epidermal growth factor recombinant protein was purchased from PeproTech (Cranbury, NJ, USA). The antibodies used in this study were as follows: anti-ERK (4695S), anti-p-ERK T202/Y204 (4370S), anti-AKT (2920S), anti-p-AKT S473 (4060S), anti-pGSK3β (ser9) (9336S), anti-GSK3β (9315S), anti-β-catenin (9562S), anti-Dkk1 (4687S) and anti-EGFR (D38B1) were purchased from Cell signaling (Danvers, MA, USA); anti-ALP (sc-365765) and anti-GAPDH (sc-47724) were purchased from Santa Cruz Biotechnology (Dallas, TX, USA); anti-pEGFR Y1068(44-788G) was purchased from Life Technology (Waltham, MA, USA); and anti-Cyclin D1(ab16663) was purchased from Abcam (Cambridge, UK).

### 4.3. Western Blotting

Cells were harvested and disrupted with cell lysis buffer (20 mM Tris, 150 mM NaCl, 1 mM EDTA, 0.5% Triton-X 100, protease inhibitor (Roche, Basel, Switzerland)), followed by sonication. Then, 30 μg of each protein sample was subjected to SDS–PAGE and transferred to a nitrocellulose membrane. After the transfer, each membrane was cropped according to the size of the target protein that needs to be hybridized with the target antibody. Then, the membranes were blocked with 5% skim milk in TBST for 30 min and then the membranes were incubated with the indicated primary antibody overnight. The membranes were labeled with horse radish peroxidase (HRP)-conjugated secondary antibodies. Chemiluminescence images were obtained using an LAS-2000 system.

### 4.4. Cell Growth Assay

Cells were seeded at a density of 5 × 10^3^ cells/well in a 96-well plate and then incubated for 24 h. Then, the medium was exchanged for DMEM containing piperonylic acid or DMSO. After 24 h incubation, 10 μL of cell counting kit-8 (Dongin LS, Seoul, Republic of Korea) solution was applied following the manufacturer’s protocol and the optical density was measured at 450 nm.

### 4.5. RNA Isolation and qRT-PCR

Cells were mixed with 600 μL of Tri-solution (Bio science Technology, Daegu, South Korea) and total RNA was extracted by adding 120 μL of chloroform. After incubation at room temperature for 10 min, samples were centrifuged at 12,000× *g* at 4 °C for 10 min. The upper phase was transferred to a fresh tube and mixed with the same volume of isopropanol. After 10 min incubation at room temperature, the samples were centrifuged again. The white RNA pellet was washed with 75% ethanol and then dissolved in DEPC-treated water. Total RNA was reverse-transcribed using the ImProm-IITM Reverse Transcription System (Promega, Madison, WI, USA) following the manufacturer’s instructions. qRT-PCR was performed using a StepOnePlus Real-Time PCR System (Thermofisher, Waltham, MA, USA) with FastStart Universal SYBR Green Master (Roche, Basel, Switzerland).

The sequences of the forward and reverse primers used in this study were as follows: Wnt5a forward 5′-TTCTCCTTCGCCCAGGTTGTAA-3′, reverse 5′-CTTCTGACATCTGAACAGGGTATTC-3′, Axin2a forward 5′-ATGATTCCATGTCCATGACG-3′, reverse 5′-CTTCACACTGCGATGCATTT-3′, lef1 forward 5′-ACAGATCACCCCACCTCTTG-3′, reverse 5′-TGATGGGAAAACCTGGACAT-3′, DKK1 forward 5′-ATTCCAACGCTATCAAGAACC-3′, reverse 5′-TTCTTGTCCTTTGGTGTGATA-3′, Egr1 forward 5′-TGACCGCAGAGTCTTTTCCT-3′, reverse 5′-TGGGTTGGTCATGCTCACTA-3′, c-Myc forward 5′-TTCGGGTAGTGGAAAACCAG-3′, reverse 5′-CAGCAGCTCGAATTTCTTCC-3′, ALP forward 5′-GGAGTATGAGAGTGACGAGAAAG-3′, reverse 5′-GAAGTGGGAGTGCTTGTATCT-3′, GAPDH forward 5′- CCAAGGAGTAAGACCCCTGG-3′, reverse 5′-AGGGGAGATTCAGTGTGGTG-3′.

### 4.6. Wound Healing Assay

The KNU201 cells were plated in a 12-well plate at 2 × 10^5^ cells per well. After 24 h incubation, each well was scraped in a straight line using a yellow pipette tip. The medium was then exchanged for DMEM containing piperonylic acid or DMSO. Images were captured at the indicated times using an Olympus phase contrast inverted microscope (Tokyo, Japan). Wound areas were quantified using Image J 1.54f software (https://imagej.net/ij/index.html, accessed on 1 January 2021) (NIH, Bethesda, MD, USA).

### 4.7. Immunocytochemistry

The KNU201 cells were plated in a 12-well plate at 2 × 10^5^ cells per wel1 on glass coverslips and incubated overnight. DMSO or 100 μM of piperonylic acid was added to each well and incubated for 4 h. After incubation, the KNU201 attached to the glass coverslips were washed once with warm PBS and fixed with 4% PFA for 40 min at room temperature (RT). After washing three times, the cells were treated with 0.5% NP-40 in PBS for 30 min and washed once. The cells were then incubated with blocking solution (5% FBS, 2.5% BSA in PBS) for 2 h at RT. Then, primary antibody (anti-β-catenin, 1:200) was incubated with the cells at 4 °C overnight. The cells were washed three times and incubated with the secondary antibody (Alexa Fluor 488 conjugate) at RT in the dark for 2 h. After washing three times, the cells were stained with Hoechst 33342 (10 μg/mL) diluted in PBS (1:2500) for 10 min in the dark and washed twice. The cells were finally mounted with fluorescence mounting medium (Dako, Glostrup, Denmark, S3023). Images were captured using an Olympus confocal microscope (FV3000) and analyzed by Image J 1.54f software (https://imagej.net/ij/index.html, accessed on 1 January 2021) (NIH, Bethesda, MD). Stained images were opened with Image J. The images were made into 8-bit. Then, the fluorescence positive area was visualized through the “threshold” tool and quantified with the “Analyze Particle” tool. For analyzing β-catenin localized to the nucleus, after selecting the Hoechst positive area with the “create selection” tool, the selected area was applied to the β-catenin positive area. After measuring the β-catenin positive area, it was divided by the number of nuclei. The number of nuclei was measured by counting the nuclei stained by Hoechst. The “Multi-point” tool was used to count the number of nuclei.

### 4.8. Human Clinical Study

The human clinical study was conducted in a double-blind, randomized manner, with 60 subjects randomly assigned to the test group or placebo group. One participant in the test group discontinued participation in the clinical test mid-study due to personal reasons (Table S2). All subjects provided consent for the publication of their photographs and personal information in an online open-access publication. Subjects were male or female with androgenetic alopecia, aged between 20 to 65 years. According to the Basic and Specific (BASP) classification, basic type included males or females diagnosed with androgenetic alopecia of M1 or higher, C1 or higher, or U1 or higher. Specific type included males or females diagnosed with androgenetic alopecia of V1 or higher, or F1 or higher. Men diagnosed with a Norwood-Hamilton classification of 2 or 2A and women diagnosed with a Ludwig classification of 1 were also included in the inclusion criteria (Table S3) [64]. The average age of the test group was 51.72 years (±6.65), and the group consisted of 24 women and 5 men. The average age of the placebo group was 47.14 years (±11.98), and the group consisted of 24 women and 6 men (Table S4). Subjects applied the formula to the test area twice a day (morning, evening), using their fingers. To evaluate the efficacy, the total hair count in the balding area was measured using a phototrichogram. To count the number of hairs in the balding area, subjects were shaved in the balding area to a length of approximately 1 cm^2^ before the first application of the formula, 16 weeks after the first application, and 24 weeks after the first application. The balding area was photographed using a hair magnification device (Folliscope 5.0, Lead M, Seoul, Republic of Korea).

### 4.9. Safety Assessments

Dermatologists evaluated the presence of erythema, edema, scaling, itching, stinging, burning, tightness, prickling, and other abnormalities by physical examination and history taking at each visit of the subjects. Dermatologists evaluated the severity of adverse reactions of the subjects and recorded the onset date and treatment given.

### 4.10. Statistical Analysis

Statistical analyses of all in vitro data were performed using GraphPad Prism version 10.0. The significance of the differences was assessed by an unpaired Student’s *t* test or one-way ANOVA followed by Tukey’s multiple comparison tests. Values of *p* < 0.05, *p* < 0.01, *p* < 0.001 and *p* < 0.0001 are indicated by *, **, *** and ****, respectively. Error bars shown in this study represent standard deviation (SD) or standard error of mean (SEM). Statistical analyses of the clinical test data were conducted using repeated measures ANOVA with the contrast test, Friedman test or Mann–Whitney U test. Values of *p* < 0.05 and *p* < 0.001 are indicated by * and ***, respectively. Delta values (the change in measurement values between the pre-application and post-application of a formula) of *p* < 0.05 and *p* < 0.01 are indicated by † and ††, respectively.

## 5. Conclusions

Piperonylic acid is a natural compound that induced the activation of the EGFR and Wnt/β-catenin signaling pathways in DPCs. Piperonylic acid activated downstream modulators of EGFR, AKT and ERK. Furthermore, piperonylic acid inhibited DKK1 expression, resulting in β-catenin accumulation in the nucleus. Piperonylic acid also stimulated DPC growth by inducing cell growth-associated factors and increased ALP expression, a marker of DPC inductivity. Furthermore, the results of the clinical study showed that the number of hairs significantly increased in the test group that applied the formula containing piperonylic acid compared with the placebo group. These findings indicate piperonylic acid as a new promising candidate for the treatment of hair loss.

## Figures and Tables

**Table 1:** Results of within-subject tests and Friedman tests. F/χ2, freedom, significance probability for each formula, before and after application. ^1^*** *p* < 0.001 by repeated measures ANOVA, ^2^ *p* by Friedman test.

	F/χ2	Freedom	Significance Probability
Test group (Piperonylic acid)	10.006	2.56	<0.001 ***^1^
Placebo group	2.643	2	0.267 ^2^

**Table 2:** Descriptive statistics and the results of within-subjects contrasts. The mean and standard deviation of the total hair count at 0, 16, and 24 weeks for each formulation. Within-subject significance probability at 16 and 24 weeks compared to baseline. * *p* < 0.05, *** *p* < 0.001 by repeated measures ANOVA with contrast test.

		Before Formula Application	16 Weeks after Formula Application	24 Weeks after Formula Application
Test group (Piperonylic acid)	Average ± Standard Deviation	163.586 ± 30.205	168.207 ± 29.493	166.000 ± 28.385
	Significance Probablity	-	<0.0001 ***	<0.03 *
Placebo group	Average ± Standard Deviation	175.100 ± 34.288	176.333 ± 35.346	174.233 ± 33.685
	Significance Probablity	-	-	-

**Table 3:** Average hair growth rate at 16 and 24 weeks after formula application.

	16 Weeks after Formula Application	24 Weeks after Formula Application
Test group (Piperonylic acid)	3.010	1.767
Placebo group	0.670	−0.403

**Table 4:** Results of Mann–Whitney U test. The change in hair count after applying each formula for 16 weeks and 24 weeks compared to 0 week and significance probability between the test group and placebo group. ^†^ *p* < 0.05, ^††^ *p* < 0.01 by Mann–Whitney U test using delta values (Δ). Delta value (Δ) is the change in hair count between the pre-application measurement and the post-application measurement.

	16 Weeks after Formula Application		24 Weeks after Formula Application	
	Test Group (Piperonylic Acid)	Placebo Group	Test Group (Piperonylic Acid)	Placebo Group
Change (Δ)	4.621	1.233	2.414	−0.867
Significance Probability	0.009 ^††^		0.047 ^†^	

## Data Availability

The datasets used and/or analyzed during the current study are available from the corresponding author on reasonable request.

## Supplementary Materials

The following supporting information can be downloaded at: https://www.mdpi.com/article/10.3390/ijms251910774/s1.

## References

[1] Morgan B.A. (2014). The dermal papilla: An instructive niche for epithelial stem and progenitor cells in development and regeneration of the hair follicle. Cold Spring Harb. Perspect. Med..

[2] Taghiabadi E., Nilforoushzadeh M.A., Aghdami N. (2020). Maintaining Hair Inductivity in Human Dermal Papilla Cells: A Review of Effective Methods. Skin Pharmacol. Physiol..

[3] Ji S., Zhu Z., Sun X., Fu X. (2021). Functional hair follicle regeneration: An updated review. Signal Transduct. Target. Ther..

[4] Driskell R.R., Clavel C., Rendl M., Watt F.M. (2011). Hair follicle dermal papilla cells at a glance. J. Cell Sci..

[5] Lin X., Zhu L., He J. (2022). Morphogenesis, Growth Cycle and Molecular Regulation of Hair Follicles. Front. Cell Dev. Biol..

[6] Oh J.W., Kloepper J., Langan E.A., Kim Y., Yeo J., Kim M.J., Hsi T.-C., Rose C., Yoon G.S., Lee S.-J. (2016). A Guide to Studying Human Hair Follicle Cycling In Vivo. J. Investig. Dermatol..

[7] Huelsken J., Vogel R., Erdmann B., Cotsarelis G., Birchmeier W. (2001). beta-Catenin controls hair follicle morphogenesis and stem cell differentiation in the skin. Cell.

[8] Tsai S.Y., Sennett R., Rezza A., Clavel C., Grisanti L., Zemla R., Najam S., Rendl M. (2014). Wnt/beta-catenin signaling in dermal condensates is required for hair follicle formation. Dev. Biol..

[9] Enshell-Seijffers D., Lindon C., Kashiwagi M., Morgan B.A. (2010). beta-catenin activity in the dermal papilla regulates morphogenesis and regeneration of hair. Dev. Cell.

[10] Hawkshaw N.J., Hardman J.A., Alam M., Jimenez F., Paus R. (2020). Deciphering the molecular morphology of the human hair cycle: Wnt signalling during the telogen-anagen transformation. Br. J. Dermatol..

[11] Kishimoto J., Burgeson R.E., Morgan B.A. (2000). Wnt signaling maintains the hair-inducing activity of the dermal papilla. Genes Dev..

[12] Choi B.Y. (2020). Targeting Wnt/beta-Catenin Pathway for Developing Therapies for Hair Loss. Int. J. Mol. Sci..

[13] MacDonald B.T., He X. (2012). Frizzled and LRP5/6 receptors for Wnt/beta-catenin signaling. Cold Spring Harb. Perspect. Biol..

[14] Liu J., Xiao Q., Xiao J., Niu C., Li Y., Zhang X., Zhou Z., Shu G., Yin G. (2022). Wnt/beta-catenin signalling: Function, biological mechanisms, and therapeutic opportunities. Signal Transduct. Target. Ther..

[15] Stamos J.L., Weis W.I. (2013). The beta-catenin destruction complex. Cold Spring Harb. Perspect. Biol..

[16] Li V.S., Ng S.S., Boersema P.J., Low T.Y., Karthaus W.R., Gerlach J.P., Mohammed S., Heck A.J., Maurice M.M., Mahmoudi T. (2012). Wnt signaling through inhibition of beta-catenin degradation in an intact Axin1 complex. Cell.

[17] Cadigan K.M., Waterman M.L. (2012). TCF/LEFs and Wnt signaling in the nucleus. Cold Spring Harb. Perspect. Biol..

[18] Doumpas N., Lampart F., Robinson M.D., Lentini A., Nestor C.E., Cantù C., Basler K. (2019). TCF/LEF dependent and independent transcriptional regulation of Wnt/beta-catenin target genes. EMBO J..

[19] Ryu Y.C., Lee D., Shim J., Park J., Kim Y., Choi S., Bak S.S., Sung Y.K., Lee S., Choi K. (2021). KY19382, a novel activator of Wnt/beta-catenin signalling, promotes hair regrowth and hair follicle neogenesis. Br. J. Pharmacol..

[20] Shin D.W. (2022). The Molecular Mechanism of Natural Products Activating Wnt/beta-Catenin Signaling Pathway for Improving Hair Loss. Life.

[21] Hawkshaw N.J., Hardman J.A., Haslam I.S., Shahmalak A., Gilhar A., Lim X., Paus R. (2018). Identifying novel strategies for treating human hair loss disorders: Cyclosporine A suppresses the Wnt inhibitor, SFRP1, in the dermal papilla of human scalp hair follicles. PLoS Biol..

[22] Kwack M.H., Kim M.K., Kim J.C., Sung Y.K. (2012). Dickkopf 1 promotes regression of hair follicles. J. Investig. Dermatol..

[23] Niida A., Hiroko T., Kasai M., Furukawa Y., Nakamura Y., Suzuki Y., Sugano S., Akiyama T. (2004). DKK1, a negative regulator of Wnt signaling, is a target of the beta-catenin/TCF pathway. Oncogene.

[24] Kwack M.H., Sung Y.K., Chung E.J., Im S.U., Ahn J.S., Kim M.K., Kim J.C. (2008). Dihydrotestosterone-inducible dickkopf 1 from balding dermal papilla cells causes apoptosis in follicular keratinocytes. J. Investig. Dermatol..

[25] Papukashvili D., Liu C., Rcheulishvili N., Xie F., Wang X., Feng S., Sun X., Zhang C., Li Y., He Y. (2023). DKK1-targeting cholesterol-modified siRNA implication in hair growth regulation. Biochem. Biophys. Res. Commun..

[26] Papukashvili D., Rcheulishvili N., Liu C., Xie F., Tyagi D., He Y., Wang P.G. (2021). Perspectives on miRNAs Targeting DKK1 for Developing Hair Regeneration Therapy. Cells.

[27] Wee P., Wang Z. (2017). Epidermal Growth Factor Receptor Cell Proliferation Signaling Pathways. Cancers.

[28] Zhang H., Nan W., Wang S., Zhang T., Si H., Wang D., Yang F., Li G. (2016). Epidermal growth factor promotes proliferation of dermal papilla cells via Notch signaling pathway. Biochimie.

[29] Choi N., Kim W.S., Oh S.H., Sung J.H. (2020). Epiregulin promotes hair growth via EGFR-medicated epidermal and ErbB4-mediated dermal stimulation. Cell Prolif..

[30] Huang H.C., Lin H., Huang M.C. (2019). Lactoferrin promotes hair growth in mice and increases dermal papilla cell proliferation through Erk/Akt and Wnt signaling pathways. Arch. Dermatol. Res..

[31] Yamane M., Seo J., Zhou Y., Asaba T., Tu S., Nanmo A., Kageyama T., Fukuda J. (2022). Effects of the PI3K/Akt signaling pathway on the hair inductivity of human dermal papilla cells in hair beads. J. Biosci. Bioeng..

[32] Kang J.I., Choi Y.K., Koh Y.-S., Hyun J.-W., Kang J.-H., Lee K.S., Lee C.M., Yoo E.-S., Kang H.-K. (2020). Vanillic Acid Stimulates Anagen Signaling via the PI3K/Akt/ beta-Catenin Pathway in Dermal Papilla Cells. Biomol. Ther..

[33] Woo H., Lee S., Kim S., Park D., Jung E. (2017). Effect of sinapic acid on hair growth promoting in human hair follicle dermal papilla cells via Akt activation. Arch. Dermatol. Res..

[34] Kang J.I., Kim M.-K., Lee J.-H., Jeon Y.-J., Hwang E.-K., Koh Y.-S., Hyun J.-W., Kwon S.-Y., Yoo E.-S., Kang H.-K. (2017). Undariopsis peterseniana Promotes Hair Growth by the Activation of Wnt/beta-Catenin and ERK Pathways. Mar. Drugs.

[35] Tomy M.J., Sharanya C.S., Dileep K.V., Prasanth S., Sabu A., Sadasivan C., Haridas M. (2015). Derivatives form better lipoxygenase inhibitors than piperine: In vitro and in silico study. Chem. Biol. Drug Des..

[36] Ranga Rao R., Tiwari A.K., Reddy P.P., Babu K.S., Ali A.Z., Madhusudana K., Rao J.M. (2009). New furanoflavanoids, intestinal alpha-glucosidase inhibitory and free-radical (DPPH) scavenging, activity from antihyperglycemic root extract of Derris indica (Lam.). Bioorg. Med. Chem..

[37] Lee D., Lim J., Woo K.C., Kim K.T. (2018). Piperonylic acid stimulates keratinocyte growth and survival by activating epidermal growth factor receptor (EGFR). Sci. Rep..

[38] Moreira K.G., Prado T.P.D., Mendes N.F., Bezerra R.d.M., Jara C.P., Lima M.H.M., de Araujo E.P. (2021). Accelerative action of topical piperonylic acid on mice full thickness wound by modulating inflammation and collagen deposition. PLoS ONE.

[39] Paul I., Bhattacharya S., Chatterjee A., Ghosh M.K. (2013). Current Understanding on EGFR and Wnt/beta-Catenin Signaling in Glioma and Their Possible Crosstalk. Genes Cancer.

[40] Lee C.H., Hung H.W., Hung P.H., Shieh Y.S. (2010). Epidermal growth factor receptor regulates beta-catenin location, stability, and transcriptional activity in oral cancer. Mol. Cancer.

[41] Hu T., Li C. (2010). Convergence between Wnt-beta-catenin and EGFR signaling in cancer. Mol. Cancer.

[42] Hwang J.H., Lee H.-Y., Chung K.B., Lee H.J., Kim J., Song K., Kim D.-Y. (2021). Non-thermal atmospheric pressure plasma activates Wnt/beta-catenin signaling in dermal papilla cells. Sci. Rep..

[43] Luo J., Chen M., Liu Y., Xie H., Yuan J., Zhou Y., Ding J., Deng Z., Li J. (2018). Nature-derived lignan compound VB-1 exerts hair growth-promoting effects by augmenting Wnt/beta-catenin signaling in human dermal papilla cells. PeerJ.

[44] Xing F., Yi W.J., Miao F., Su M.Y., Lei T.C. (2018). Baicalin increases hair follicle development by increasing canonical Wnt/beta-catenin signaling and activating dermal papillar cells in mice. Int. J. Mol. Med..

[45] Kwack M.H., Ahn J.S., Kim M.K., Kim J.C., Sung Y.K. (2012). Dihydrotestosterone-inducible IL-6 inhibits elongation of human hair shafts by suppressing matrix cell proliferation and promotes regression of hair follicles in mice. J. Investig. Dermatol..

[46] Kwack M.H., Jang Y.J., Won G.H., Kim M.K., Kim J.C., Sung Y.K. (2019). Overexpression of alkaline phosphatase improves the hair-inductive capacity of cultured human dermal papilla spheres. J. Dermatol. Sci..

[47] McElwee K.J., Kissling S., Wenzel E., Huth A., Hoffmann R. (2003). Cultured peribulbar dermal sheath cells can induce hair follicle development and contribute to the dermal sheath and dermal papilla. J. Investig. Dermatol..

[48] Poch B., Gansauge F., Schwarz A., Seufferlein T., Schnelldorfer T., Ramadani M., Beger H.G., Gansauge S. (2001). Epidermal growth factor induces cyclin D1 in human pancreatic carcinoma: Evidence for a cyclin D1-dependent cell cycle progression. Pancreas.

[49] Shtutman M., Zhurinsky J., Simcha I., Albanese C., D’Amico M., Pestell R., Ben-Ze’Ev A. (1999). The cyclin D1 gene is a target of the beta-catenin/LEF-1 pathway. Proc. Natl. Acad. Sci. USA.

[50] Philpott M.P., Kealey T. (1994). Effects of EGF on the morphology and patterns of DNA synthesis in isolated human hair follicles. J Investig. Dermatol..

[51] Boisvert W.A., Yu M., Choi Y., Jeong G.H., Zhang Y.-L., Cho S., Choi C., Lee S., Lee B.-H. (2017). Hair growth-promoting effect of Geranium sibiricum extract in human dermal papilla cells and C57BL/6 mice. BMC Complement. Altern. Med..

[52] York K., Meah N., Bhoyrul B., Sinclair R. (2020). A review of the treatment of male pattern hair loss. Expert Opin. Pharmacother..

[53] McClellan K.J., Markham A. (1999). Finasteride: A review of its use in male pattern hair loss. Drugs.

[54] Gupta A.K., Venkataraman M., Talukder M., Bamimore M.A. (2022). Finasteride for hair loss: A review. J. Dermatolog. Treat.

[55] Suchonwanit P., Thammarucha S., Leerunyakul K. (2019). Minoxidil and its use in hair disorders: A review. Drug Des. Dev. Ther..

[56] Rossi A., Cantisani C., Melis L., Iorio A., Scali E., Calvieri S. (2012). Minoxidil use in dermatology, side effects and recent patents. Recent Pat. Inflamm. Allergy Drug Discov..

[57] Zhang H., Nan W., Wang S., Zhang T., Si H., Yang F., Li G. (2016). Epidermal Growth Factor Promotes Proliferation and Migration of Follicular Outer Root Sheath Cells via Wnt/beta-Catenin Signaling. Cell Physiol. Biochem..

[58] Bai T., Liu F., Zou F., Zhao G., Jiang Y., Liu L., Shi J., Hao D., Zhang Q., Zheng T. (2017). Epidermal Growth Factor Induces Proliferation of Hair Follicle-Derived Mesenchymal Stem Cells Through Epidermal Growth Factor Receptor-Mediated Activation of ERK and AKT Signaling Pathways Associated with Upregulation of Cyclin D1 and Downregulation of p16. Stem Cells Dev..

[59] Tripurani S.K., Wang Y., Fan Y.-X., Rahimi M., Wong L., Lee M.-H., Starost M.F., Rubin J.S., Johnson G.R. (2018). Suppression of Wnt/beta-catenin signaling by EGF receptor is required for hair follicle development. Mol. Biol. Cell.

[60] Bichsel K.J., Hammiller B., Trempus C.S., Li Y., Hansen L.A. (2016). The epidermal growth factor receptor decreases Stathmin 1 and triggers catagen entry in the mouse. Exp. Dermatol..

[61] Richardson G.D., Bazzi H., Fantauzzo K.A., Waters J.M., Crawford H., Hynd P., Christiano A.M., Jahoda C.A.B. (2009). KGF and EGF signalling block hair follicle induction and promote interfollicular epidermal fate in developing mouse skin. Development.

[62] Lin Y., Liu C., Zhan X., Wang B., Li K., Li J. (2019). Jagged1 and Epidermal Growth Factor Promoted Androgen-Suppressed Mouse Hair Growth In Vitro and In Vivo. Front. Pharmacol..

[63] Xing Y., Ma X., Guo H., Deng F., Yang J., Li Y. (2016). Wnt5a Suppresses beta-catenin Signaling during Hair Follicle Regeneration. Int. J. Med. Sci..

[64] Lee W.S., Ro B.I., Hong S.P., Bak H., Sim W.-Y., Kim D.W., Park J.K., Ihm C.-W., Eun H.C., Kwon O.S. (2007). A new classification of pattern hair loss that is universal for men and women: Basic and specific (BASP) classification. J. Am. Acad. Dermatol..

