# Supplementary material for: Piperonylic Acid Promotes Hair Growth by Activation of EGFR and Wnt/β-Catenin Pathway
Source: Int J Mol Sci. 2024 Oct 7;25(19):10774. doi: 10.3390/ijms251910774 (PMC11476903; doi:10.3390/ijms251910774)
Supplement: Supplementary file 1 [file ijms-25-10774-s001.zip › ijms-3226182-supplementary.pdf]

Supplementary figure 1. Piperonylic acid activates EGFR downstream modulator in pDPCs

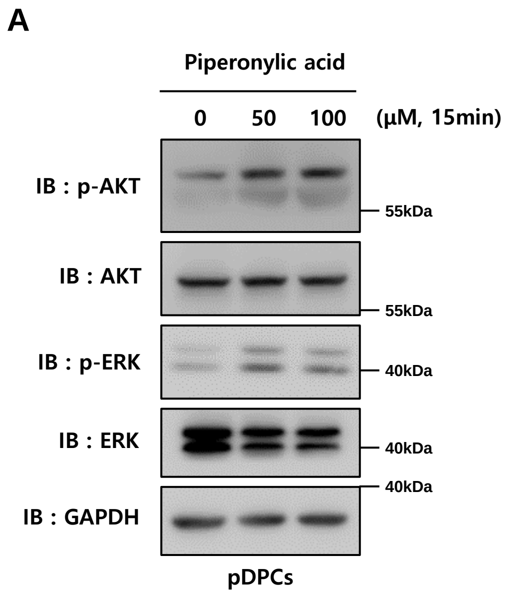

Supplementary figure 1
(A) Piperonylic acid activates EGFR downstream modulator in pDPCs. pDPCs were cultured in serum free media for 18h. After incubation, cells were treated with indicated concentration of piperonylic acid for 15mins. Cell lysates were analyzed by immunoblotting.

Supplementary figure 2. Piperonylic acid activates Wnt/β-catenin pathway.

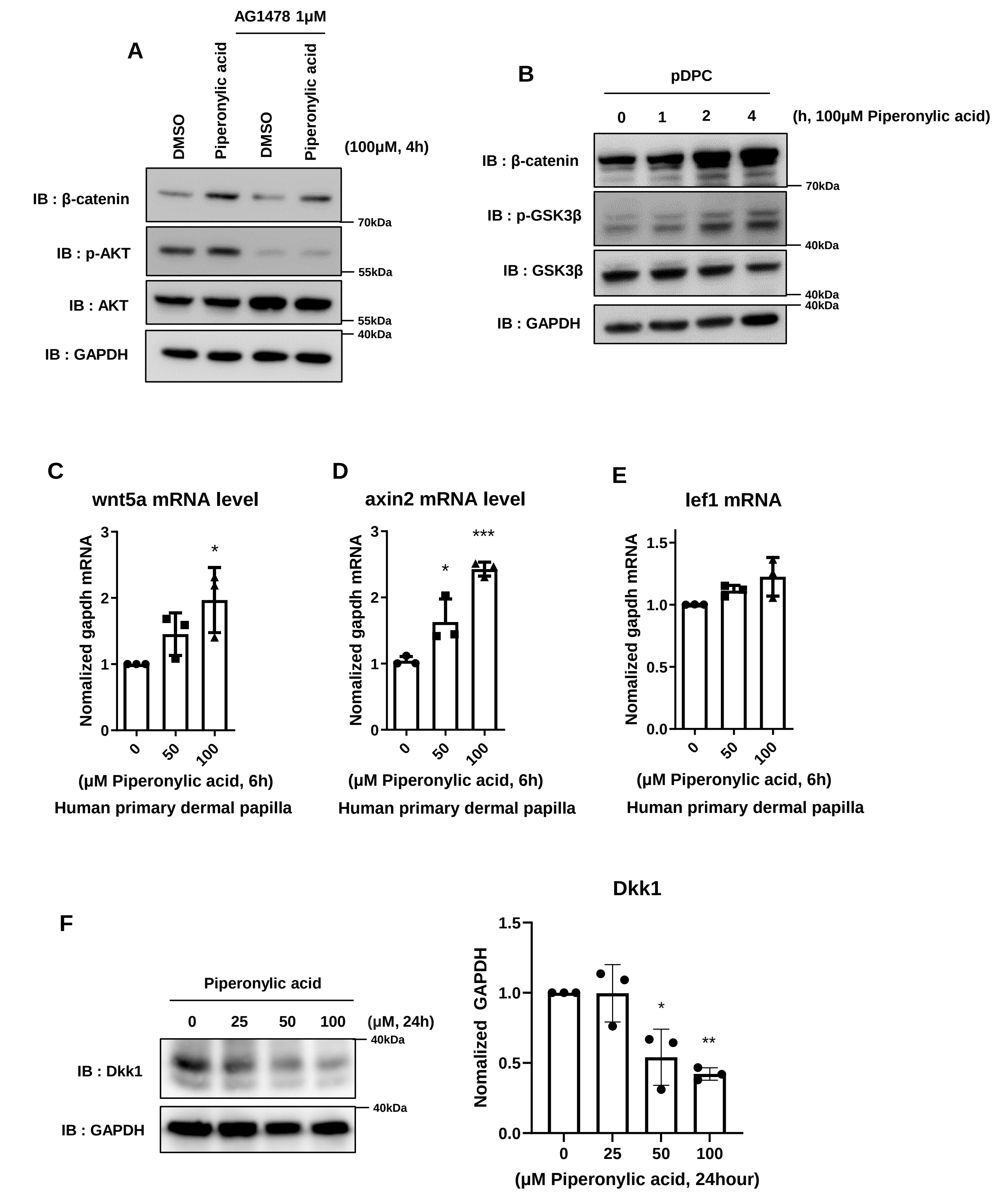

Supplementary figure 2. Piperonylic acid activates Wnt/β-catenin pathway.

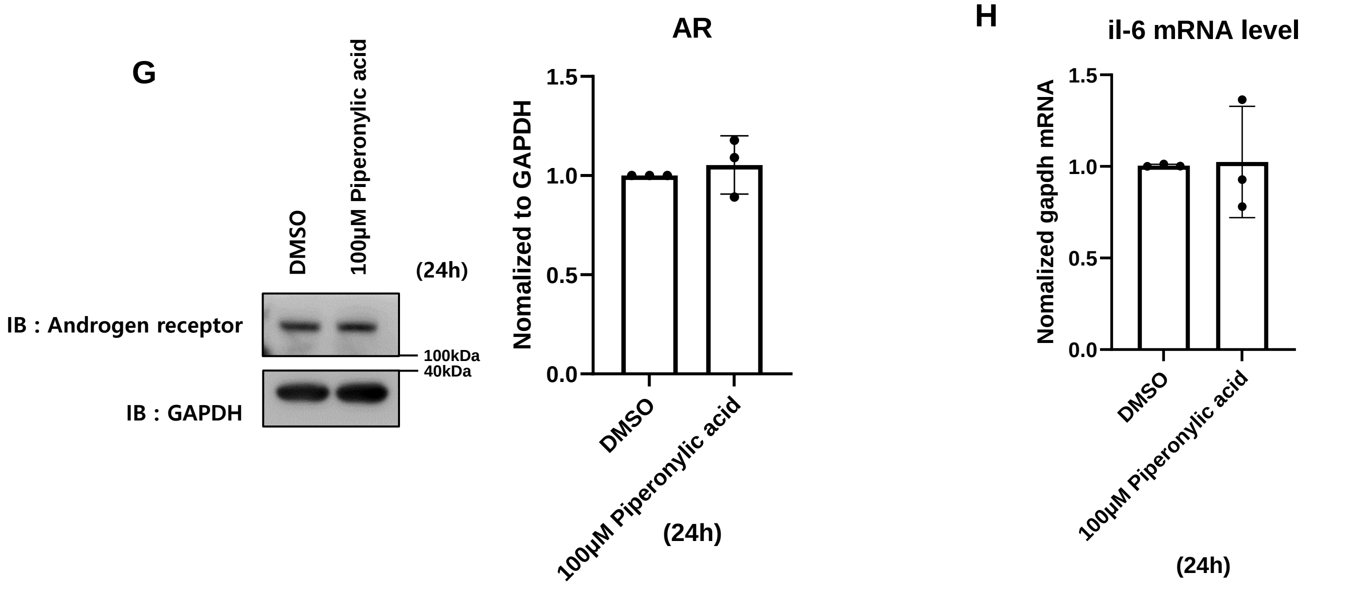

Supplementary figure 2. Piperonylic acid activates Wnt/β-catenin pathway.

(A) Piperonylic acid induced accumulation of β-catenin is not inhibited by treatment of AG1478. DPCs were treated with indicated drugs simultaneously for 4hours. Cell lysates were analyzed by immunoblotting.

(B) Piperonylic acid activates Wnt/β-catenin signaling pathway in pDPCs. DPCs were treated with 100μM piperonylic acid for indicated time points. Cell lysates were analyzed by immunoblotting.

(C-E) Piperonylic acid promotes the expression of Wnt-related genes in pDPCs. DPCs were treated with indicated concentration of piperonylic acid for 6 hours. wnt5a(C), axin2a(D) and lef1 mRNA were examined by real time PCR and nomalized to gapdh mRNA. Values represent means ± standard deviation (SD) (n=3, independent experiments) *p<0.05, ***p< 0.005, by Tukey's multiple comparison test.

(F) Piperonylic acid decrease the Dkk1 in DPCs. DPCs were treated with indicated concentration of piperonylic acid for 24 hours. Cell lysates were analyzed by immunoblotting. Dkk1 levels were normalized to GAPDH. Values represent means ± standard deviation (SD), (n=3, independent experiments). *p<0.05, **p<0.01 by Tukey's multiple comparison test.

(G) Piperonylic acid has no effect on AR expression. DPCs were treated with DMSO or 100μM piperonylic acid for 24h. Cell lysates were analyzed by immunoblotting. Dkk1 levels were normalized to GAPDH. Values represent means ± standard deviation (SD), (n=3, independent experiments).

(H) Piperonylic acid has no effect on the expression of il-6, a target gene of AR. DPCs were treated with DMSO or 100μM piperonylic acid for 24h. il-6 mRNA were examined by real time PCR and nomalized to gapdh mRNA. Values represent means ± standard deviation (SD) (n=3, independent experiments)

Supplementary Table 1. Basic information of research subjects

	Test group (Piperonylic acid)	Placebo group
Resistered subjects	30	30
Dropped-out subjects	1	0
Completed subjects	29	30
Average age (Standard deviation)	51.72(6.65)	47.14(11.98)
Sex	Woman(24), Man(5)	Woman(24), Man(6)

Supplementary Table 1. Basic information of research subjects

Supplementary Table 2. Age distribution of subjects

	20-29	30-39	40-49	50-59	60-69	Total
Test group (Piperonylic acid)	-	2	7	17	3	29
Placebo group	4	1	10	11	4	30

Supplementary Table 2. Age distribution of subjects.

Supplementary Table 3. Preliminary tests of homogeneity

Z	Significance Probability
-1.191	0.234

Supplementary Table 3. Preliminary tests of homogeneity. Z value is a test statistics that measures the difference between two independent samples.

Supplementary Table 4. Androgenic alopecia classification

Test group				Placebo group			
Number	BASP	N-H	Ludwig	Number	BASP	N-H	Ludwig
2	-	-	Ludwig1	1	-	-	Ludwig1
4	-	-	Ludwig1	3	-	-	Ludwig1
6	-	-	Ludwig1	5	-	-	Ludwig1
8	-	-	Ludwig1	7	-	-	Ludwig1
11	-	-	Ludwig1	9	-	-	Ludwig1
13	M1F1	-	-	10	M1F1	-	-
14	-	-	Ludwig1	12	-	-	Ludwig1
17	-	-	Ludwig1	15	-	-	Ludwig1
18	M1F1	-	-	16	M1F1	-	-
20	-	-	Ludwig1	19	-	-	Ludwig1
23	-	-	Ludwig1	21	-	-	Ludwig1
24	M1F2	-	-	22	M1F1	-	-
26	-	-	Ludwig1	25	-	-	Ludwig1
29	-	-	Ludwig1	27	-	-	Ludwig1
32	-	-	Ludwig1	28	M1F1	-	-
34	-	-	Ludwig1	31	-	-	Ludwig1
37	-	-	Ludwig1	33	-	-	Ludwig1
39	-	-	Ludwig1	35	-	-	Ludwig1
41	-	-	Ludwig1	36	M1F2	-	-
42	M1F1	-	-	38	-	-	Ludwig1
45	-	-	Ludwig1	40	-	-	Ludwig1
47	-	-	Ludwig1	43	M1F2	-	-
48	M1V1	-	-	44	-	-	Ludwig1
50	-	-	Ludwig1	46	-	-	Ludwig1
52	-	-	Ludwig1	49	-	-	Ludwig1
54	-	-	Ludwig1	51	-	-	Ludwig1
56	-	-	Ludwig1	53	-	-	Ludwig1
58	-	-	Ludwig1	55	-	-	Ludwig1
60	-	-	Ludwig1	57	-	-	Ludwig1
				59	-	-	Ludwig1

Supplementary Table 4. Androgenic alopecia classification.
